# Strain Specific Resistance to Murine Scrapie Associated with a Naturally Occurring Human Prion Protein Polymorphism at Residue 171

**DOI:** 10.1371/journal.ppat.1002275

**Published:** 2011-09-29

**Authors:** James F. Striebel, Brent Race, Kimberly D. Meade-White, Rachel LaCasse, Bruce Chesebro

**Affiliations:** Laboratory of Persistent Viral Diseases and Rocky Mountain Laboratories, National Institute of Allergy and Infectious Diseases, Hamilton, Montana, United States of America; University of Edinburgh, United Kingdom

## Abstract

Transmissible spongiform encephalopathies (TSE) or prion diseases are neurodegenerative disorders associated with conversion of normal host prion protein (PrP) to a misfolded, protease-resistant form (PrPres). Genetic variations of prion protein in humans and animals can alter susceptibility to both familial and infectious prion diseases. The N171S PrP polymorphism is found mainly in humans of African descent, but its low incidence has precluded study of its possible influence on prion disease. Similar to previous experiments of others, for laboratory studies we created a transgenic model expressing the mouse PrP homolog, PrP-170S, of human PrP-171S. Since PrP polymorphisms can vary in their effects on different TSE diseases, we tested these mice with four different strains of mouse-adapted scrapie. Whereas 22L and ME7 scrapie strains induced typical clinical disease, neuropathology and accumulation of PrPres in all transgenic mice at 99-128 average days post-inoculation, strains RML and 79A produced clinical disease and PrPres formation in only a small subset of mice at very late times. When mice expressing both PrP-170S and PrP-170N were inoculated with RML scrapie, dominant-negative inhibition of disease did not occur, possibly because interaction of strain RML with PrP-170S was minimal. Surprisingly, in vitro PrP conversion using protein misfolding cyclic amplification (PMCA), did not reproduce the in vivo findings, suggesting that the resistance noted in live mice might be due to factors or conditions not present in vitro. These findings suggest that in vivo conversion of PrP-170S by RML and 79A scrapie strains was slow and inefficient. PrP-170S mice may be an example of the conformational selection model where the structure of some prion strains does not favor interactions with PrP molecules expressing certain polymorphisms.

## Author Summary

Transmissible spongiform encephalopathies (TSE) or prion diseases are infectious fatal neurological diseases that affect many mammals, including humans. In these diseases a misfolded form of host prion protein (PrP) leads to brain degeneration and death. The genetic code of PrP in individual animals or humans has minor variations, which in some cases are associated with altered susceptibility to disease. In humans a variation at residue 171 (N171S) has been found in people mainly of African descent. However, due to the low incidence of the variation and difficult accessibility of these individuals, studies of prion diseases in these populations have not been carried out. Therefore, to create a laboratory animal model to study the effect of this variation on prion diseases, we generated transgenic mice expressing the mouse version of the human PrP variation at residue 171. We then studied the susceptibility of these mice to 4 strains of mouse-adapted scrapie. In our experiments these transgenic mice were uniquely resistant to two scrapie strains, but showed high sensitivity to two others. This resistance appeared to be related to a slow or inefficient generation of the aggregated disease-associated form of PrP in these mice, and was not duplicated using in vitro assays. In summary, transgenic mice expressing this variant PrP provide an interesting model to study differences among prion strains and their interactions with PrP in vivo.

## Introduction

TSE or prion diseases are transmissible neurodegenerative diseases occurring in a variety of mammalian species including domestic and wild animals as well as humans [1]. Examples include scrapie in sheep, chronic wasting disease (CWD) in cervids and bovine spongiform encephalopathy (BSE) in cattle. Human diseases include sporadic and variant Creutzfeldt-Jakob disease (sCJD and vCJD) and familial diseases such as Gerstmann–Sträussler–Scheinker syndrome (GSS), Fatal Familial Insomnia (FFI) and familial CJD. A hallmark of prion diseases is the conversion of the normal protease-sensitive host prion protein (PrPsen) into a misfolded partially protease-resistant form (PrPres) which may be in part responsible for the generation of the disease [2]. PrP expression is required for prion disease, and PrP knockout mice are resistant to both infection and disease [3].

PrP gene polymorphisms occur naturally in many species. In both sheep and in mice such polymorphisms have been found to influence susceptibility to scrapie infection and disease. For example, sheep expressing Alanine, Arginine, Arginine at positions 136, 154 and 171 (A_136_R_154_R_171_) respectively, are highly resistant to most strains of sheep scrapie [4], [5], [6]. However, recent observations indicate that sheep with the A_136_R_154_R_171_ genotype can be susceptible to atypical scrapie (Nor98) suggesting that resistance might be strain specific [7], [8]. Similarly in inbred mice there are two known PrP alleles (Prnp^a^ and Prnp^b^) which are characterized by amino acid differences at PrP residues 108 and 189 [9], [10], [11]. Mice with Prnp^a^ have short incubation times with one set of scrapie strains and prolonged incubation times with another set, while mice with Prnp^b^ show an opposite pattern of incubation times with these same scrapie strains [12], [13]. Thus there is a different strain-specific pattern of susceptibility associated with each of these Prnp genotypes.

In humans, the methionine vs. valine polymorphism at PrP residue 129 appears to influence the clinical phenotype of familial prion disease associated with the D178N PrP mutation [14], [15], as well as susceptibility to sCJD, vCJD and kuru [16], [17], [18], [19], [20]. In addition, the human polymorphism E219K may be associated with resistance to sCJD [21], but does not appear to correlate with resistance to vCJD in humans or mouse models [22], [23]. The human PrP polymorphism, G127V, was recently shown to be associated with resistance to kuru [24]. Other naturally occurring human polymorphisms, such as G142S and N171S, have not been tested for prion disease susceptibility [25].

N171S is a PrP polymorphism found in Sub-Saharan Africans, Jamaicans and Sardinians [26]. The rarity of this polymorphism and the fact that it is present mainly in geographical regions with limited CJD surveillance make it difficult to detect possible associations with prion disease. Therefore, in order to initiate laboratory studies of the possible effects of this polymorphism on TSE diseases and possibly also other CNS disorders, we generated transgenic mice expressing mouse PrP-170S, the mouse homolog of human PrP-171S (Table 1).

**Table 1:** Comparison of PrP residues in humans, mice and transgenic mice generated in the present study.

Human residue	165	166	167	168	169	170	171	172	173	174	175
Mouse residue	164	165	166	167	168	169	**170**	171	172	173	174
Human	P	M	D	E	Y	S	**N/S** a	Q	N	N	F
Mouse	P	V	D	Q	Y	S	**N**	Q	N	N	F
Transgenic mice^b^	P	V	D	Q	Y	S	**S**	Q	N	N	F

a- Naturally occurring polymorphism at human PrP residue 171 (see [26] for review).

b- In the present study mouse residue 170 was mutated to generate 3 lines of transgenic mice (Tg330,Tg340, and Tg290). Human PrP residue 171 is homologous to mouse PrP residue 170.

This approach of using transgenic mice expressing mouse or hamster PrP with human PrP mutations and/or polymorphic residues at homologous sites has been taken in numerous previous studies of prion diseases. These include important studies of models of Gerstmann-Sträussler-Scheinker syndrome (GSS) [27], [28], [29], [30], [31], [32], FFI [33] and familial CJD [34], [35], as well as a non-infectious neurodegenerative disease associated with expression of prion protein with a nine octapeptide insertion [36]. In addition, transgenic mice expressing mouse PrP with cervid PrP residues associated with a “rigid loop” in PrP have also revealed interesting in vivo pathogenic effects [37], [38].

Because PrP variations in animals and humans can have different effects on different TSE strains, we tested our mice with four different scrapie strains which have been maintained in continuous mouse passage for many years. In these studies PrP-170S expressing mice were highly susceptible to scrapie strains 22L and ME7, but were markedly resistant to scrapie strains RML and 79A. In contrast, control mice were susceptible to all four strains. This in vivo strain-specific influence on scrapie susceptibility was not reproduced in cell-free in vitro PrP conversion studies, suggesting that in vivo conditions not replicated in our in vitro system were required. PrP-170S transgenic mice appear to be an interesting new model to study the interactions between TSE agent strains and PrP.

## Materials and Methods

### Ethics statement

All mice were housed at the Rocky Mountain Laboratories (RML) in an AAALAC-accredited facility and experimentation followed NIH RML Animal Care and Use Committee approved protocols (NIH/RML Protocol #2007-31). This study was carried out in strict accordance with the recommendations in the Guide for the Care and Use of Laboratory Animals of the National Institutes of Health.

### Generation of transgenic mice

To study the effect of the human N171S PrP polymorphism (Ref SNP#rs16990018) we constructed transgenic mice expressing serine instead of asparagine at mouse PrP residue 170 (homologous to human PrP residue 171) (Table 1). The mutation was made at residue 170 in mouse PrP using a cDNA clone of mouse PrP, p1-5 (E48-16) [39] using oligonucleotides 2077U (5′- CCA GTG GAT CAG TAC AGC AGC CAG AAC AAC TTC GTG C -3′) and 2078L (5′- GCA CGA AGT TGT TCT GGC TGC TGT ACT GAT CCA CTG G -3′) with a site directed mutagenesis kit (Stratagene/Agilent, Santa Clara, CA). The plasmid with the mutation was recloned, and the mutation was confirmed by sequencing. A DNA fragment from PshAI to SfoI with PrP sequences containing the mutation was excised, purified and religated into a subclone, p44-3 (E58-6) [39], [40], derived from the pHGPrP half-genomic clone [41] by digesting with AgeI and SfoI to remove a portion of the PrP ORF and replacing this segment with an oligonucleotide polylinker containing these and other sites. Upstream sequences previously excised between two BamHI sites to remove an unwanted SfoI site were replaced by digestion with adjacent sites NotI and BspEI and the original 6.2kb fragment from PHGPrP was reinserted. The resulting plasmid, p188-6, was digested with NotI and SbfI to separate mouse sequences from bacterial plasmid sequences, and the fragment containing the mouse sequences was used to inoculate C57BL/6 mouse eggs to generate transgenic mice expressing PrP-170S [39]. Of the five transgenic founding lines produced, three lines (Tg330, Tg340 and Tg290) were selected for experimentation. These transgenic lines were hemizygous for the transgene and homozygous for Prnp, the gene encoding normal mouse PrP (MoPrP). The Prnp gene encoding PrP-170N in C57BL/6 mice was removed by serial backcrossing to C57BL/10Sn mice with a knocked-out Prnp gene (B10 PrP-/-) derived from the original Edinburgh Prnp knockout mouse as previously described [39], [42]. Transgenic lines were maintained as transgene heterozygotes by crossing to C57BL/10 PrP-/- mice and selection of transgene positive mice by PCR analysis of tail DNA.

Genotyping was conducted using standard PCR reactions as previously described [39]. Briefly, detection of the modified knock-out version of Prnp and the neo cassette in the PrP-/- mice was accomplished using primers RK1 and Mut217 as previously described [43]. These primers yielded a C57BL/10 MoPrP product of approximately 700 bp and a PrP-/- neomycin cassette gene product of approximately 1700bp. Detection of the half-genomic PrP transgene, expressing the N170S PrP construct, was accomplished using primers pE2+ and Mut217 as described previously [43]. PCR products were visualized via electrophoresis in a 2% agarose gel.

### Laboratory animals

All mice were housed at the Rocky Mountain Laboratories (RML) in an AAALAC-accredited facility and experimentation followed NIH RML Animal Care and Use Committee approved protocols (NIH/RML Protocol #2007-31). This study was carried out in strict accordance with the recommendations in the Guide for the Care and Use of Laboratory Animals of the National Institutes of Health. Mice were bred and genotyped at RML. For use as controls, weanling C57BL/10Hsd mice were obtained from Harlan Sprague Dawley, Madison, WI. Transgenic tga20 mice were obtained from EMMA (Munich, Germany) [41].

### Scrapie inoculations and animal observations

Mice were injected intracerebrally (i.c.) with 50μl of a 1% (wt/vol) dilution of brain homogenate pools from C57BL mice terminally ill from 22L, RML, 79A or ME7 scrapie. Two stocks of RML scrapie (RML-06, RML-81) were used. Titers of scrapie stocks were determined in previous i.c. endpoint titration experiments and were as follows; 22L = 2.5×10^9^, ME7 = 4.0×10^8^, RML-06 =  3.2×10^8^, RML-81 =  4.8×10^8^, 79A = 1.6×10^8^ (units =  50% infectious dose (ID_50_)/gm of brain). Brain homogenates were diluted for inoculation in phosphate buffered balanced saline (PBBS) pH 7.2, supplemented with 2% fetal bovine serum (Hyclone, Logan, UT).

Observations were made daily to assess clinical signs of scrapie disease, which included ataxia, altered gait, wasting, kyphosis, hind limb weakness, aimless wandering, somnolence, immobility and leg clasping reflex. Mice with clinical signs were euthanized and brain tissue was analyzed for PrPres by immunoblotting. Mice with both clinical signs and PrPres by immunoblot were defined as diseased, and the day of euthanasia was recorded as the incubation period in the data presented. Uninoculated control transgenic mice were followed up to 700 days of age and showed no clinical signs of scrapie differing from normal signs of senescence. Several uninoculated mice were analyzed by histopathology after euthanasia at 465 days of age, and brain tissue had no detectable grey matter vacuolation or abnormal PrPres deposition.

Statistical analysis of data in the coexpression experiment was done by a one way ANOVA with Dunnett's multiple comparison test using GraphPad Prism software.

### Immunoblot analysis

Tissue samples from mice were analyzed for PrPres and PrPsen by immunoblot. Brain homogenates (20%w/v) were prepared in 10 mM Tris-HCl [pH 7.4] using a mini-beadbeater (Biospec products, Bartlesville, OK). All homogenates were sonicated for 1 min using a Vibracell cup-horn sonicator (Sonics, Newtown, NJ) as previously described [40]. To test for PrPres, samples were proteinase K treated as follows; 20 µl of a 20% (w/v) tissue homogenate was adjusted to 100 mM Tris HCl (pH 8.3), 1% Triton X-100,1% sodium deoxycholate and 50 µg/ml proteinase K (PK), in a total volume of 31 µl. Tubes were mixed and incubated for 45 minutes at 37°C. The reaction was stopped by adding 2 µl of 100 mM Pefabloc (Roche Diagnostics, Indianapolis, Indiana) and placed on ice for 5 min. Samples tested for PrPsen were treated with proteinase inhibitors: 10 µM leupeptin, 1 µM pepstatin A, and 7 µg/ml aprotinin. PrPsen samples were not treated with PK. An equal volume of 2X Laemmli sample buffer (Biorad, Hercules, CA) was added to both PrPsen and PrPres samples, and then tubes were boiled 5 minutes. Samples were frozen at −20°C until the day of analysis, when samples were thawed, reboiled for 5 minutes and then electrophoresed on a 16% Tris-Glycine SDS-PAGE gel (Invitrogen, Carlsbad,CA) and blotted to PVDF membranes (Biorad) using a 7 minute transfer, program 3 (P3) on an iBlot (Invitrogen) device. Immunoblots were blocked in a solution of 2.5% milk (Biorad) in 0.1 M Tris, 1.5M NaCl and 0.05% Tween20, for 1 hour. Then blots were probed with D13 anti-PrP monoclonal antibody [44], (InPro, San Francisco, CA) at a final concentration of 0.2 µg/ml, followed by a peroxidase-conjugated anti-human IgG secondary antibody (Sigma, St. Louis, MO) at a final dilution of 1∶5000 in the blocking buffer described above. Bands were detected using enhanced chemiluminescence substrate (ECL) as directed by manufacturer (GE Healthcare, Pittsburgh, PA).

### Histology and immunohistochemistry

For histopathological analysis mice were deeply anesthetized and euthanized by cervical dislocation. Tissues were removed and placed in 3.7% phosphate-buffered formalin for 3 to 5 days before dehydration and embedding in paraffin. Serial 5 µm sections were cut using a standard Leica microtome, placed on positively charged glass slides and dried overnight at 43°C. Slides were then deparaffinized using standard procedures. Slides were stained with hematoxylin and eosin and analyzed for pathological changes. Immunohistochemical detection of PrPres using DAB chromogen (DAB Map kit; Ventana Medical Systems, Tucson, AZ.) was done as follows: antigen retrieval and staining were performed using the Ventana automated Discovery XT stainer. PrP antigens were exposed by incubation in CC1 buffer (Ventana) containing Tris-Borate-EDTA, pH 8.0 for 20 minutes at 95°C. Staining for PrP was done using human anti-mouse PrP monoclonal antibody D13 at a dilution of 1∶500 at 37°C for 2 hours, followed by a biotinylated anti-human IgG at 1∶500 (Jackson ImmunoResearch, West Grove, PA.), and avidin-horseradish peroxidase with DAB as chromogen. Slides were examined and photomicrographs were taken observed using an Olympus BX51 microscope and Microsuite FIVE software.

### Protein misfolding cyclic amplification (PMCA) assay

For preparation of normal brain homogenates containing PrPsen to be used as the substrate for PMCA reactions healthy transgenic mice expressing either PrP-170S (Tg330) or PrP-170N (Tga20) [41] were deeply anesthetized and then perfused with PBS containing 5 mM EDTA. Brains were removed and homogenized in a beadbeater at a concentration of 20% (w/v) in PMCA conversion buffer (PBS containing 4 mM EDTA, 1% Triton X-100 and complete mini-protease inhibitor cocktail (Roche), sterilized by filtration with a 0.2 µm filter) then cooled on ice for 10–15 min. Homogenates were diluted with PMCA conversion buffer to 10% (w/v) then clarified by a brief 1500 X g spin [45]. Supernatants were stored at −80° C in 1 ml aliquots until used in PMCA reactions.

Brain homogenates used as seeds for the PMCA reactions came from clinically sick 22L or RML-infected C57BL/6 mice. Brains were homogenized at a 20% (w/v) concentration using 0.1 M Tris pH 7.4, then diluted in the same buffer to a final concentration of 10% (w/v) prior to storage at −80°C. Scrapie-positive brain homogenates were used to “seed” normal brain homogenate in the following manner. A 10% scrapie-positive brain homogenate (seed) was added to normal brain homogenate (substrate) at desired dilutions and then these master mixes were aliquoted into multiple 0.2ml reaction tubes (GeneMate, ISCBioexpress, Kaysville, Utah). One of these tubes was frozen as an unsonicated control and the remaining tubes were repeatedly sonicated and incubated as described below. Tubes were positioned in a plastic tube rack PMCA adapter (Misonix, Farmingdale, NY) and placed on the rim of a microplate horn of a Misonix Model 3000 microsonicator so that the 50 µl samples were immersed in the sonicator bath. The microplate horn was covered with a plastic lid to minimize evaporation from the water bath. The sonicator was located inside an incubator set to 37°C and was programmed to perform cycles, each consisting of a 40 second pulse of sonication set at 60% maximum followed by a 30 min incubation. Forty-eight cycles (i.e. 24 h) constituted one round of PMCA.

PMCA reaction tubes were removed from the sonicator and vortexed and 10 µl of the 50 µl volume was sampled and mixed with 10 µl PK at 100 µg/ml for a final PK concentration of 50 µg/ml. Samples were maintained at 37°C in a water bath for one hour. The PK digestions were halted with 2 µl of 100 mM Pefabloc (Roche Diagnostics) and placed on ice for 5 min. An equal volume of 2X Laemmli sample buffer (Biorad, Hercules, CA) was added to the PMCA samples, and then tubes were boiled 5 minutes. Samples were frozen at −20°C until the day of analysis, when samples were thawed, and reboiled for 5 minutes. PMCA products were visualized by SDS page gel electrophoresis and immunoblotting as described above for brain tissues, with the following exceptions. Gels were blotted to HyBond ECL nitrocellulose membranes (GE Healthcare Life Sciences) instead of PVDF membrane. Immunoblots were blocked in Near-Infrared Fluorescence Western Blotting Blocking Buffer (Rockland Immunochemicals Inc., Gilbertsville, PA) and PBS mixed in equal parts. Primary antibody was D13 1∶100 diluted supernatant derived from CHO cells expressing the D13 antibody construct [44]. These cells were obtained from R. Anthony Williamson, The Scripps Research Institute, La Jolla, CA. The secondary antibody was IRDye800CW-conjugated goat-anti-mouse IgG (LiCor, Lincoln, NE) diluted at 1∶10,000. Both antibodies were diluted in the blocking buffer described above with the addition of 0.2% Tween 20. Finally, bands were detected using an Odyssey near-infrared fluorescence scanner (LiCor). Groups were compared using a Mann-Whitney test with GraphPad Prism software.

## Results

### Generation, breeding and PrP expression of transgenic mice expressing mouse PrP-170S

To study the effect of the human PrP polymorphism, N171S, on susceptibility to prion disease we generated transgenic mice expressing mouse PrP-170S, the mouse homolog of the human PrP-171S (Table 1). Five founder lines were produced on the C57BL/6 background, and these were crossed serially to C57BL/10 mice homozygous for the Edinburgh version of the PrP null gene (Prnp-/-) [42]. Three lines of the transgenic mice (Tg290, Tg330, and Tg340) with the Prnp-/- gene, and hemizygous for the PrP-170S transgene, were obtained and used for further study.

**Figure 1:**
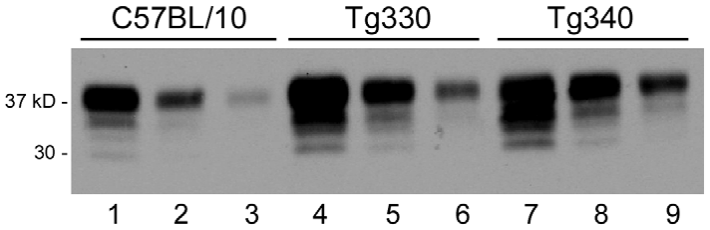
Immunoblot detection of PrPsen from C57BL/10 and transgenic mice. Blot was developed using anti-PrP monoclonal antibody D13 and enhanced chemiluminescence exposure for 1 minute. Lanes 1-3 show brain PrP-170N expression level in a C57BL/10 mouse, lanes 4-6 and lanes 7-9 show brain PrP-170S expression in Tg330 and Tg340 mice. For each mouse, three lanes beginning on the left were loaded with 0.5, 0.25 and 0.125 mg tissue equivalents, respectively. Comparison of these groups of samples suggested 2-3-fold greater PrP expression in the transgenic mice versus C57BL/10 mice. Several different individual transgenic mouse brains were also studied, and results confirmed the data presented here. Tg330 and Tg340 also expressed detectable levels of PrPsen in kidney, cecum, adrenal gland, heart, spleen and skeletal muscle (immunoblots not shown).

Because PrPsen expression is known to influence scrapie incubation period [41], [46], we determined PrPsen levels in brain by immunoblot. Both Tg330 and Tg340 mice expressed PrPsen at levels 2 to 3-fold higher than were seen in non-transgenic control C57BL/10 mice (Prnp+/+) which express PrP-170N (Figure 1). Tg290 expressed approximately 10-fold lower PrP levels than did control mice (data not shown). All three strains of PrP-170S mice were next tested for susceptibility to scrapie infection.

### Resistance to scrapie strains RML and 79A in PrP-170S transgenic mice

**Figure 2:**
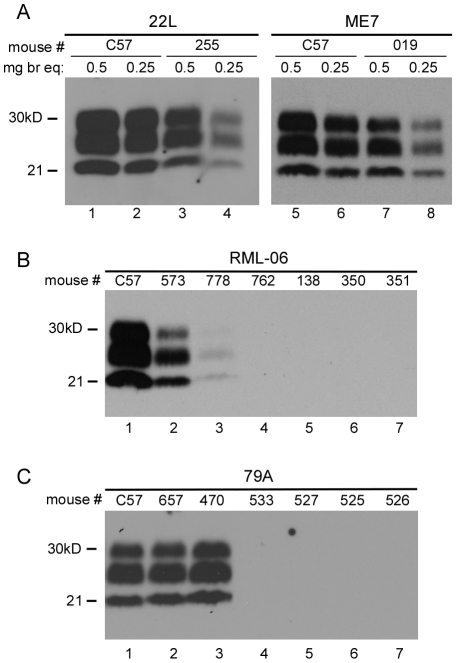
Immunoblot analysis of PrPres in scrapie-infected mice. (A) Panel A shows immunoblots of brain homogenates tested using both 0.5mg and 0.25mg brain equivalents per lane. PrPres levels in clinical Tg330 mice (#255 and #019) were slightly lower than levels seen in clinical C57BL/10 mice. All C57BL/10 and Tg330 mice inoculated with 22L and ME7 scrapie were positive for PrPres by western blot at the time of clinical disease. In panels B and C, lanes 1 and 2 had 0.5 mg brain equivalents and other lanes had 1.0mg. (B) RML (stock 06) inoculations. Of fifteen transgenic mice tested, two (#573 and 778) were positive for PrPres at 451 and 518 days respectively. Four of the 13 PrPres-negative mice are also shown. RML (stock 81) inoculation of 14 mice resulted in none positive for PrPres by immunoblot (not shown). (C) 79A inoculations. Of fourteen transgenic mice tested, two mice (#657 and 470) were positive for PrPres at 439 and 545 dpi respectively. Four of the 12 PrPres-negative mice are also shown.

Four strains of scrapie were used to test the influence of the N170S polymorphism on susceptibility to TSE disease. In Tg330 and Tg340 mice, the 22L and ME7 scrapie strains produced 100% incidence of typical fatal prion disease with clinical signs similar to control C57BL/10 mice (Table 2). Shorter incubation periods were observed in the Tg330 and Tg340 mice than in C57BL/10 mice, probably due to higher PrPsen expression levels in the transgenic mice. The diagnosis of scrapie was confirmed by the detection of PrPres in brain by immunoblotting. Brain PrPres levels in transgenic mice were slightly less than those in control mice, possibly due to shorter incubation periods (Figure 2A). In Tg290 mice inoculation with scrapie strain 22L produced disease in 6 of 8 mice at an average incubation period of 615 dpi (data not shown). This lower incidence of disease and slower tempo appeared to be related to the low PrPsen expression in Tg290 mice, and this line was not studied further.

**Table 2:** Susceptibility of transgenic and C57BL/10 mice to four strains of scrapie.

	Mouse strains					
	C57BL/10		Tg330		Tg340	
Scrapie strains^a^	Diseased/total	Incubation period^b^	Diseased/total	Incubation period^b^	Diseased/total	Incubation period^b^
22L	11/11	141±5	18/18	109±9	5/5	128±7
ME7	8/8	153±6	8/8	99±3	11/11	101±4
RML-06	10/10	179±8	0/8	na^c^	2/7	451, 518
RML-81	8/8	146±6	0/7	na	0/7	na
79A	8/8	132±6	2/7	439, 545	0/7	na

a =  For strain RML, two different stocks, RML-06 and RML-81 were studied.

b =  Incubation period is average days post-inoculation to disease +/− standard deviation.

c  =  not applicable (mice did not develop disease during 545-603 days of observation).

Unexpected results were observed upon inoculation of RML and 79A scrapie strains. Whereas control C57BL/10 mice were uniformly susceptible to these two strains, transgenic Tg 330 and Tg340 mice were quite resistant. Only two mice inoculated with RML and two mice inoculated with 79A had clinical disease and PrPres detectable by immunoblot (Figure 2), and all four occurred at late times ranging from 439-545dpi (Table 2).

**Figure 3:**
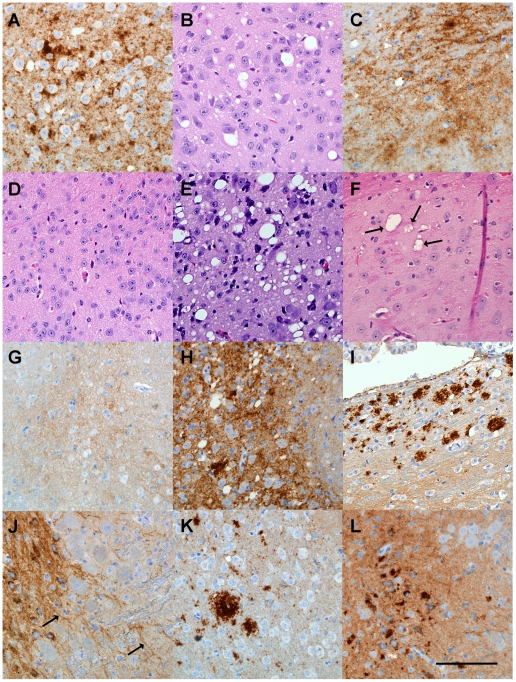
Histopathological analysis of brain tissue from transgenic mice inoculated with various scrapie strains. (A) (B) ME7-inoculated transgenic mouse with clinical signs at 98 dpi showing cerebral cortex with extensive PrPres deposition by D13 staining (A) and vacuolation by H&E staining (B). (C) 22L-inoculated transgenic mouse with clinical signs at 123dpi showing PrPres deposition in hypothalamus. (D) H&E stained area of pons from control uninoculated 465 day old mouse. (E) Vacuolation of pons in 79A-inoculated transgenic mouse with clinical disease at 545dpi. (F) Several pleomorphic and multiloculated vacuoles (arrows) seen by H&E staining in anterodorsal area of thalamus of RML-inoculated mouse at 603dpi. Vacuoles were not seen in other brain regions of this mouse. Mouse was negative for clinical signs and for PrPres by immunoblot. (G) D13 anti-PrP staining of pons from a 465 day old uninoculated control mouse. Note light brown PrPsen staining in background. (H) D13-stained pons from a 79A-inoculated mouse positive for clinical signs and PrPres by immunoblot at 545 dpi. Localized areas with similar stained aggregates were also seen in the thalamus, hypothalamus and hippocampus of this mouse. (I) D13-positive PrPres in anterodorsal area of thalamus of an RML-inoculated mouse negative for both PrPres by immunoblot and clinical signs at 603 dpi. Deposits were not detected in other brain regions. Light staining in lower part of panel is PrPsen which was also seen in uninoculated mice. (J) Positive D13 staining around cells and along linear structure of extracellular space or possibly axons (arrows) in pons from an RML-inoculated mouse negative for both PrPres by immunoblot and clinical signs at 545dpi. No other brain regions had detectable staining. (K) Stained PrPres aggregates in anterodorsal thalamus from 79A-inoculated mouse negative for clinical signs and for PrPres by immunoblot at 545dpi. Similar aggregates were also seen in the pons. (L) Stained PrPres aggregates in pons from a 79A-inoculated mouse negative for clinical signs and for PrPres by immunoblot at 545 dpi. PrPres was detected only in a small area of the pons. Diffuse staining in background is PrPsen. Scale bar is 100 µm and applies to all panels.

Brains were also examined microscopically for pathology and presence of PrPres. In Tg mice infected with strains ME7 or 22L, at the time of clinical disease vacuolation and PrPres deposition were widespread in many brain regions (Figure 3A,B,C) similar to non-transgenic control mice (not shown). In contrast, after infection with strains 79A or RML, Tg mice with clinical signs or PrPres detectable by immunoblot showed localized vacuolation and PrPres deposition (Figures 3E and 3H) mostly limited to the thalamus, hippocampus and pons. In addition, ten RML or 79A-infected Tg mice, who were negative for PrPres by immunoblot, had subclinical infection as shown by brain PrPres deposits mainly localized to the vestibular nuclei in the pons or to the anterodorsal region of the thalamus detected at 545-603dpi (Figure 3I,J,K,L). In these same areas gray matter vacuolation was either absent or minimal (Figure 3F). Detection of these subclinical mice provided evidence of a higher incidence of infection than was shown by standard clinical observation and analysis of brain PrPres by immunoblotting. Perhaps these mice should be considered preclinical as they might have developed clinical disease if allowed to survive for a longer time. These results suggested that infection of Tg PrP-170S mice by RML or 79A scrapie occurred at slow and reduced levels, and this appeared to account for the rare presence of clinical signs in this experiment.

### In vitro generation of RML and 22L PrPres using protein misfolding cyclic amplification (PMCA)

Our in vivo experiments showed a dramatic difference in the tempo and levels of PrPres generation in brains of Tg330 and Tg340 mice after infection with scrapie strains RML and 79A compared to strains 22L and ME7. Possibly a delay in PrP conversion and subsequent accumulation of PrPres might account for our results. Therefore to test PrP conversion under in vitro conditions we used the PMCA cell-free system [47]. As a source of PrPsen, brain homogenates derived from either Tga20 mice expressing PrP-170N or Tg330 mice expressing PrP-170S were used, and these reactions were initiated by seeding with PrPres from PrP-170N mice infected with scrapie strains 22L or RML.

**Figure 4:**
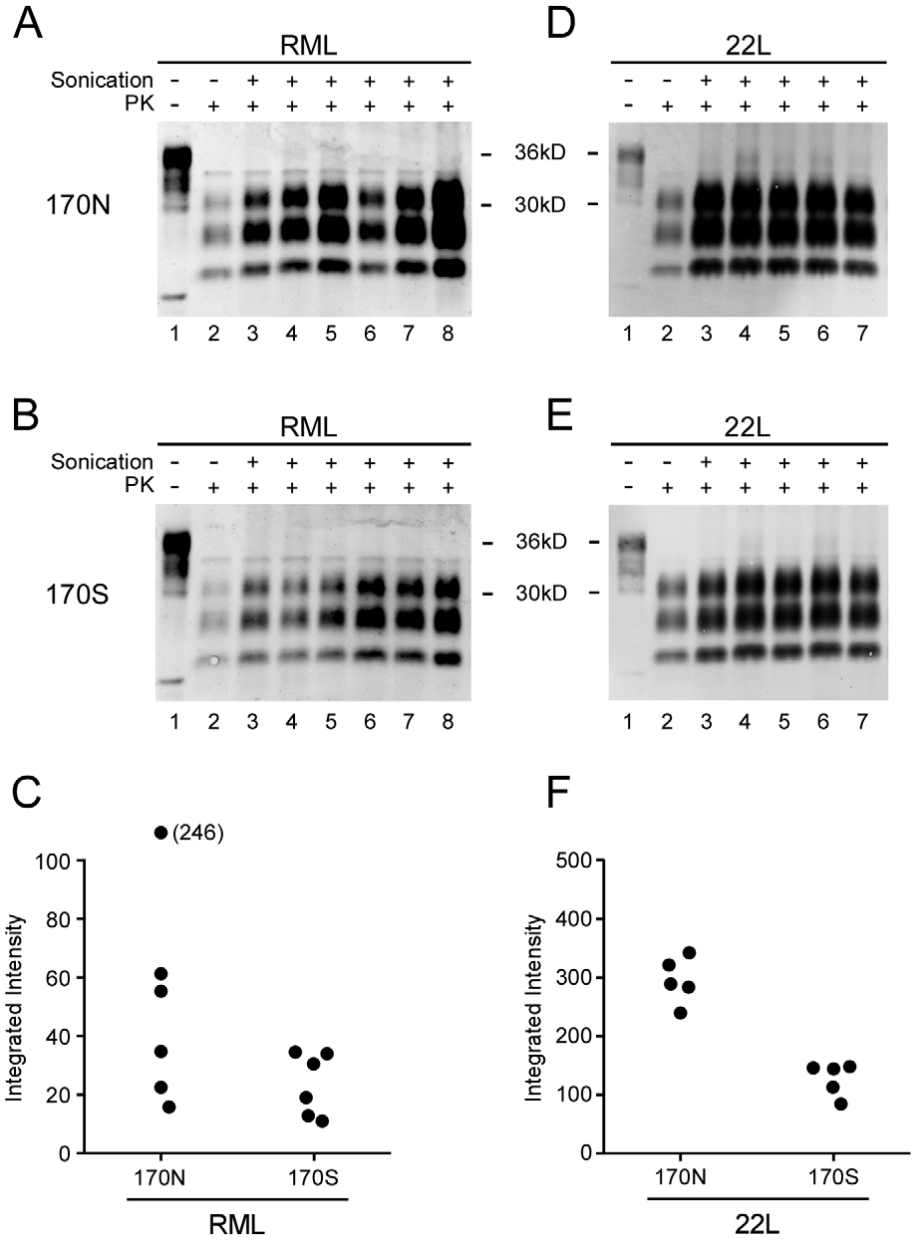
PMCA reactions comparing PrP-170N and PrP-170S as PrPsen substrates. Panels A, B, D, and E show immunoblots of PMCA products visualized on the LiCOR Odyssey Scanner. RML (A and B) or 22L (D and E) scrapie-infected brain homogenates diluted 1∶400 were used to seed two different brain homogenate substrates: 170N from Tga20 mice (A and D) and 170S from Tg330 mice (B and E). For all gels, lane 1 contains unseeded Tga20 brain homogenate (5 µg brain equivalent of Tga20 brain homogenate), which was used as a control to show differences in detection sensitivity (gain setting) on the Odyssey scanner. For example, gain was increased for panels A and B compared to D and E because of the lower signal obtained with the RML seed in both 170N and 170S substrates. Other lanes contain 3.5 µl of PK-digested sonicated reaction product (lanes 3–8) or unsonicated freeze–control (lane 2). Amplification is evident by comparison of the unsonicated lanes with corresponding sonicated lanes. Results shown are representative of several independent experiments. Panels C and F show quantitation of PrPres as integrated intensity, i.e. the quantitative measure of protein detected by the scanner, from each lane of experiments shown in A, B, D, and E (using LyCor Odyssey scanner and software). Closed circles represent values for sonicated samples minus values for unsonicated freeze-control samples. Groups were compared by a 2-tailed nonparametric Mann Whitney test. For the 22L seed, the 170N and 170S substrates were significantly different (P<0.01), and for the RML seed, the 170N and 170S were not significantly different (P = 0.093).

Surprisingly, RML PrPres seed generated PrPres conversion with both PrP-170N and PrP-170S brain homogenates. Quantification of PrPres produced in the PMCA revealed slightly greater amounts of product in the PrP-170N reactions, but these differences were not statistically significant (Figure 4A, 4B and 4C). However, the positive conversion of PrP-170S by RML PrPres indicated that there was not a basic inability of RML scrapie PrPres to convert PrP-170S. This was in contrast to the slow conversion in vivo requiring over 400 days when Tg330 and Tg340 mice were inoculated with RML scrapie (Figures 2 and 3).

In similar PMCA reactions 22L PrPres seed also gave higher PrPres generation using PrP-170N compared to PrP-170S substrate. These differences were statistically different (Figure 4D, 4E, 4F), and might in part be due to the higher PrP expression in substrates from tga20 versus Tg330 mice. In summary, comparison of PrP-170N and PrP-170S mice by seeding with PrPres from RML or 22L gave no evidence that in vitro conversion of PrP-170S by RML PrPres was abnormal as detected by PMCA.

### Infectivity studies in mice expressing both PrP-170S and PrP-170N

In numerous previous studies expression of two different PrP alleles from the same or different species has been shown to reduce the level of prion infection as well as the incidence and tempo of disease [22], [48], [49]. The resistance of the Tg330 and Tg340 mice to RML and 79A scrapie prompted us to test whether the presence of both PrP-170S and PrP-170N in the same mouse would result in lower disease incidence or increased incubation times.

To investigate this question, 22L or RML scrapie was inoculated into mice expressing two PrP alleles, one allele of the PrP-170S transgene and one allele of the mouse Prnp gene expressing PrP-170N. Mice expressing only one allele of PrP-170N (Prnp+/−) were inoculated as controls. With the 22L strain, mice expressing both 170S and 170N PrP had significantly faster incubation times (106–115dpi) than mice expressing only 170N (259 dpi) (Table 3). Therefore interference between these PrP variants did not seem to occur. The decrease in incubation period seen when both PrP alleles were expressed could be explained if the PrP-170S variant could contribute to a more rapid incubation time, as would be predicted by the susceptibility of the original Tg330 and Tg340 mice to 22L infection.

**Table 3:** Effect of coexpression of 170N and 170S PrPsen on prion disease after inoculation with scrapie strains 22L or RML.

			22L		RML	
Mouse Strain	PrPsen^a^	Genotype	Diseased/total	Incubation period^b^	Diseased/total	Incubation period
Prnp+/-	0.5X	170N/-c	11/11	259±5	8/8	299±70
Tg330+/−Prnp+/−	2.5-3.5X	170N/S	5/5	106±14^d^	5/5	231±37^e^
Tg340+/−Prnp+/−	2.5-3.5X	170N/S	5/5	115±15^d^	3/4	244±56^e^

a  =  expression of PrPsen relative to C57BL/10 Prnp+/+ mouse.

b  =  incubation period is average days post inoculation +/− standard deviation to disease after inoculation with 22L or RML (stock RML-81) scrapie as described in the methods.

c  =  Prnp+/− mice have only one PrP allele and this allele expresses 170N.

d  =  p<0.001 compared to Prnp+/− mice using a 1-way ANOVA with Dunnett's multiple comparison test.

e  =  After RML infection, neither Tg330 nor Tg340 were significantly different from Prnp+/− mice when compared using a 1-way ANOVA with Dunnett's multiple comparison test (P = 0.1457).

In the case of strain RML, the expression of both alleles was associated with a decrease in incubation time compared to expression of PrP-170N alone, but these differences were not statistically significant (Table 3). Therefore, the more highly expressed PrP-170S protein appeared to contribute very little towards accelerating the disease tempo in these mice, and also there was no evidence for interference. This outcome might be predicted from the very low susceptibility of the PrP-170S transgenic mice to RML scrapie infection (Table 2). In summary, the PrP-170S variant appeared to be “neutral” during the infection by RML scrapie, showing no interference with the PrP-170N in the co-expression experiment.

## Discussion

In the present experiments the influence of a normal human prion protein gene allelic variation, N171S, on prion disease susceptibility was studied in a mouse system using transgenic mice expressing the mouse homolog, N170S. Susceptibility to prion disease induced by mouse scrapie strains, 22L and ME7, was identical in control mice expressing PrP-170N versus transgenic mice expressing PrP-170S. In contrast, after inoculation with scrapie strains, RML or 79A, mice expressing PrP-170S were markedly resistant compared to control mice expressing PrP-170N. Of the 43 transgenic mice inoculated with these two scrapie strains, disease occurred only after 439 days and this was seen in only 4 mice. An additional 10 mice had subclinical infection after 545–603 days, as shown by detection of PrPres by IHC, however the remaining 29 mice had no evidence of clinical signs or PrPres accumulation in brain after observation up to 603 days. These results demonstrated that mice expressing PrP-170S were highly resistant to infection by strains RML and 79A, but this resistance was strain-specific since there was no resistance to two other strains (ME7 and 22L).

**Figure 5:**
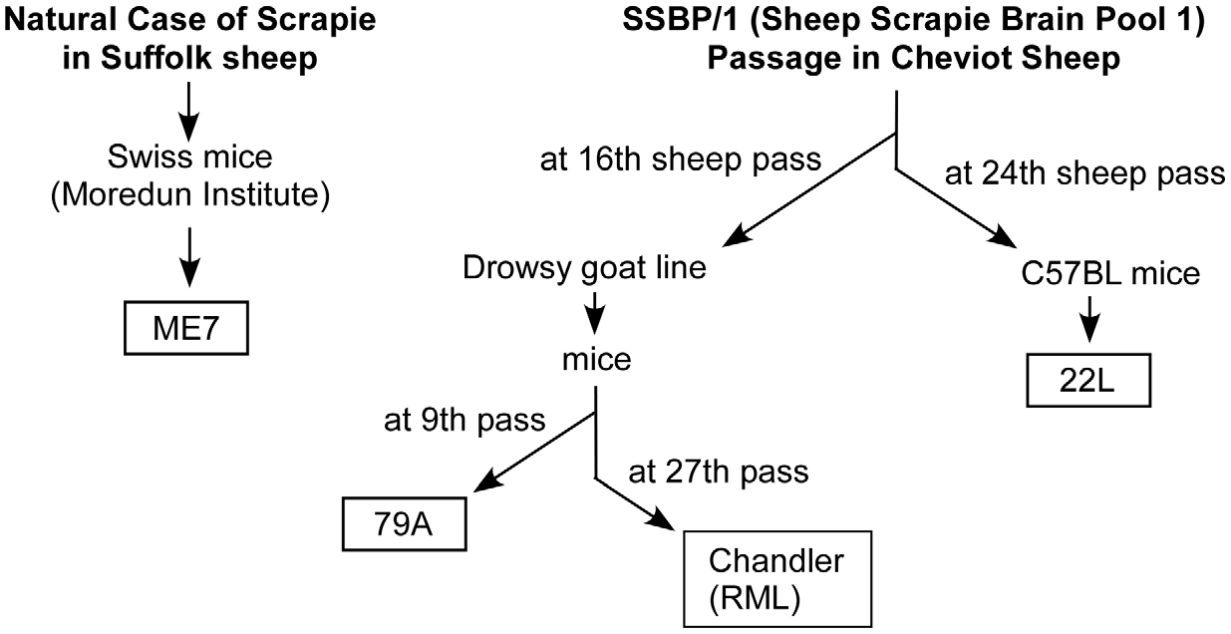
History of scrapie strains used. The derivations and passage history of strains ME7 [50], 79A [51], Chandler (RML) [52] and 22L [51] are shown. Chandler (RML) and 79A are both derived from the “drowsy goat” line [82], and therefore are likely to be more closely related to each other than to strains ME7 or 22L.

One explanation for the scrapie strain-specific differences seen in our experiments may lie in the origins of the scrapie strains used (Figure 5). Strain ME7 was the result of a passage of natural scrapie in Suffolk sheep directly to mice and therefore was not related to other scrapie strains[50]. On the other hand, 22L, RML and 79A share a common origin in that they were all derived from the Moredun Institute's sheep scrapie brain pool 1(SSBP/1)[51]. However, there were major differences in the passage history of each of these 3 strains, and strains 79A and RML were more closely related to each other than to strain 22L [51], [52] (Figure 5). This may explain the similar resistance pattern of strains 79A and RML in PrP-170S mice.

Although the biochemical explanation for scrapie strains in general remains a mystery at this time, it has been hypothesized that PrPres from each strain adopts a slightly different conformation which is conferred on successive PrPsen molecules as they are converted to PrPres [53]. Accordingly, our current data might be explained by the conformational selection model of prion strains and species barriers [54], [55] which was first developed to explain prion strain differences involving the Sup35 protein in yeast [56], [57], [58]. This model suggests that only a subset of all possible PrPres conformations is compatible with any individual PrP primary structure. Thus incompatibility between the infecting prion strain and the host PrP would result in a transmission barrier [54]. For example, mouse PrP-170S might easily assume the conformations required by strains ME7 or 22L, but might be less adept at assuming the conformations associated with strains RML or 79A. In practice most mutations studied lead to altered incubation periods [30], [31], but when strong species barriers exist, either no transmission or low level subclinical cross-species transmission has been observed [59], [60], [61].

Another possible explanation for our results is that in vivo conversion of PrP-170S by RML or 79A strains might generate PrPres with a lower than usual stability. This might be due to either high dissociation of PrPres into smaller oligomers or increased susceptibility of PrPres to catabolic degradation. However, we were not able to detect any evidence for the presence of PrPres with increased susceptibility to proteinase K in PrP-170S transgenic mice infected by either RML or 79A scrapie strains (data not shown).

Effects of PrP amino acid variations on prion disease species and strain transmission barriers in some cases appear to correlate with differences in PrP conversion. For example, sheep homozygous for PrP with V_136_R_154_Q _171_ or A_136_R_154_Q _171_ show opposite patterns of susceptibility to scrapie strains SSBP1 and CH1641 [62], [63], and in vitro generation of PrPres in PMCA reactions agreed with the in vivo resistance observed [64]. However, when we tested PrP conversion in vitro using PMCA, both 22L and RML PrPres were able to seed the generation of PrPres derived from PrP-170S (Figure 4). This conversion of PrP-170S by RML scrapie was in contrast to the ineffective production of disease and slow and low PrPres generation in PrP-170S mice infected with RML scrapie. Biological and biochemical differences between conditions in brain tissue of live mice and PMCA test tube reactions might account for the discrepancies between our in vivo and in vitro results. Similar discrepancies between PrPres generation by PMCA in vitro and clinical susceptibility have been noted previously, and in some cases strong in vivo transmission barriers between species have been easily overcome by using PMCA with minor alterations in conditions [65]. Thus PrP conversion by PMCA would appear to be less selective than in vivo infection by TSE agents.

Interestingly, the N171S polymorphism in humans (homologous to the N170S change in our Tg330 and Tg340 mice) occurs near other PrP residues implicated in influencing PrP structure and folding as well as susceptibility to prion diseases in animals. For example, alterations in PrP folding in vitro have been noted after mutations homologous to human residues 168 [66] and 170 [67]. At the structural level, PrP mutation at human residue 170 (S170N) appears to create a stabilized loop structure located near residues 165-175 [68], and this change may influence susceptibility to CWD and other prion agents [37], [38]. However, others have reported no effect of S170N on species-specific PrP conversion in vitro in a mouse-hamster system [69], [70], [71]. In contrast, resistance of rabbits to prion disease appears to be associated with a serine at human PrP residue 174 [72]. Similarly, the sheep polymorphism at residue 171 (human residue 168) is important in the resistance of sheep to classical scrapie strains [6], [73], [74]. Possibly the N171S polymorphism examined in the present study might be able to modulate prion disease because of its location near the PrP loop structure and other nearby influential residues.

In humans, familial prion diseases have been associated with PrP mutations in the near vicinity of the PrP loop structure, i.e. Q160X [75], Y163X [76], D178N [77] and V180I [78]. The N171S polymorphism is not by itself associated with familial prion disease [26]. However, a previous study identified a family with an unusual psychiatric disorder associated with PrP N171S [79]. More recently, in an African-American family with unusual psychiatric signs and sleep abnormalities preceding onset of familial CJD, disease was linked to expression of a PrP molecule containing both PrP N171S and D178N mutations [80]. Interestingly this is the first family with African ancestry where the D178N mutation has been detected, as the 12 previously reported families were of European or Japanese descent [78], [80]. This family might be an example where the N171S polymorphism altered the clinical disease signs when expressed in combination with a known pathogenic PrP mutation. It remains unclear whether this effect is mediated by a direct influence of these mutations on PrP misfolding or whether indirect effects involving other non-PrP molecules might also play a role. Nevertheless, the fact that the N171S polymorphism is present in healthy populations of humans [81], suggests that N171S is likely non-pathogenic by itself and that there may even be a selective advantage for maintaining its presence in human genomes.
